# *Mesorhizobium koreense* sp. nov., Isolated from Soil

**DOI:** 10.4014/jmb.2404.04026

**Published:** 2024-06-24

**Authors:** Hyosun Lee, Dhiraj Kumar Chaudhary, Dong-Uk Kim

**Affiliations:** 1 Department of Biological Science, College of Science and Engineering, Sangji University, Wonju 26339, Republic of Korea; 2 Department of Microbiology, Pukyong National University, Busan 48513 Republic of Korea

**Keywords:** *Mesorhizobium koreense* sp. nov. soil, *Phyllobacteriaceae*; *Pseudomonadota*, phylogenetics

## Abstract

An aerobic, Gram-stain-negative, catalase-positive, rod-shaped, and motile bacteria, designated as a strain WR6^T^ was isolated from soil in Republic of Korea. Strain WR6^T^ grew at temperatures of 10–37°C, at pH of 5.0–9.0, and at NaCl concentrations of 0–3.0% (w/v). Phylogenetic and 16S rRNA gene nucleotide sequence analysis confirmed that strain WR6^T^ affiliated to the genus *Mesorhizobium*, with the nearest relative being *Mesorhizobium waimense* ICMP 19557^T^ (98.5%). The genome of strain WR6^T^ was 5,035,462 bp with DNA G+C content of 62.6%. In strain WR6^T^, Q-10 was sole ubiquinone; summed feature 8 (C_18:1_*ω*7*c* and/or C_18:1_*ω*6*c*) and C_19:0_ cyclo *ω*8*c* were predominant fatty acids; and diphosphatidylglycerol, phosphatidylglycerol, phosphatidylmethylethanolamine, phosphatidylcholine, and phosphatidylethanolamine were major polar lipids. Based on these polyphasic taxonomic data, strain WR6^T^ represents a novel species in the genus *Mesorhizobium*. Accordingly, we propose the name *Mesorhizobium koreense* sp. nov., with the type strain WR6^T^ (=KCTC 92695^T^ =NBRC 116021^T^).

## Introduction

*Mesorhizobium* bacteria constitute a diverse array of soil-dwelling microorganisms essential for nitrogen fixation, increasing soil nitrogen availability, enhancing crop yield, and diminishing reliance on chemical fertilizers [1]. Additionally, *Mesorhizobium* species are renowned for their capacity to synthesize various enzymes and secondary metabolites [2, 3]. Reports indicate that *Mesorhizobium* species can release cellulase, proteases, lipases, and exopolysaccharides, rendering them applicable in bioremediation, bioconversion, and biopharmaceuticals [4-6]. Moreover, *Mesorhizobium* strains contribute substantially to producing biofuel and biopolymers [7, 8]. These attributes underscore the promising role of *Mesorhizobium* strains in sustainable crop production, biotechnological innovation, and industrial applications. Consequently, exploring *Mesorhizobium* strains from diverse environments is imperative for harnessing their potential as valuable industrial bioresources.

The *Mesorhizobium* genus belongs to the family *Phyllobacteriaceae* within the phylum *Pseudomonadota* and was proposed by Jarvis *et al*. in 1997, designating *Mesorhizobium loti* as the type species [9]. *Mesorhizobium* members are aerobic, Gram-negative, non-spore-forming, and rod-shaped bacteria widely recognized for their nitrogen fixation capabilities. The primary respiratory quinone system is ubiquinone-10, and the DNA G+C content ranges between 59.0%–64.0% [10-12]. As of May 2024, the genus *Mesorhizobium* comprises 63 valid species with accurate names (https://lpsn.dsmz.de/genus/mesorhizobium). These bacteria exhibit habitat versatility and are commonly isolated from root nodules of various plants [12-15]. Beyond nodular environments, *Mesorhizobium* species have also been found in seawater, soil, sediment, coal bed water, and sludge [10, 16-18]. In this study, we conducted a taxonomic investigation of strain WR6^T^ isolated from a soil sample. The presented taxonomic data confirm that strain WR6^T^ represents a novel species within the genus *Mesorhizobium*.

## Materials and Methods

### Isolation of Strain

Strain WR6^T^ was isolated from a soil sample collected from the Republic of Korea (36°33'15.1"N 126°54'05.0"E). A standard dilution plating technique with R2A media (MB Cell) was employed for the isolation. Subsequently, the plates were incubated aerobically at 25°C for one week. Following multiple streaking on R2A agar, pure colonies of strain WR6^T^ were obtained and preserved at 4°C until taxonomic analysis was completed. For long-term preservation, strain WR6^T^ was stored in R2A broth supplemented with 20% (v/v) glycerol stock at -80°C.

### 16S rRNA Gene Sequence and Phylogenetic Analysis

Genomic DNA from strain WR6^T^ was extracted using the HiGene Genomic DNA Prep Kit (BIOFACT, Republic of Korea). PCR was performed to amplify the 16S rRNA gene using the 27F and 1492R primer set [19]. Subsequently, the amplified gene was sequenced and analyzed following standard protocols [20]. The closest phylogenetically related taxa were identified by analyzing the 16S rRNA gene sequences on the EzBioCloud database server [21]. Phylogenetic trees based on the 16S rRNA gene and two housekeeping genes (gyrase subunit B, *gyrB*; and RNA polymerase subunit B, *rpoB*) were generated using MEGA X [22] through maximum–likelihood (ML) [23] and neighbor-joining (NJ) algorithms [22]. The tree topologies were estimated using the bootstrap resampling method with 1,000 replications [23], and the evolutionary distances were calculated using Kimura's two-parameter model [24].

### Genome Analysis

The genome sequencing of strain WR6^T^ was conducted using the PacBio sequencing technique, and assembly of the raw sequences was performed with NextDenovo v. 2.4.0. Quality assessment of the genome data was carried out using the ContEst16S algorithm [25], CheckM [26], and Blast-N tool [27]. Subsequently, the genome sequence was annotated using the prokaryotic genome annotation pipeline (PGAP) [28] and the rapid annotation subsystem technology (RAST) server [29]. The DNA G+C content was determined from the genome sequence data. Genomic relatedness between strain WR6^T^ and the closest species was assessed using digital DNA–DNA hybridization (dDDH) [30] and average nucleotide identity (ANI) [31] web-based tools. Gene clusters for putative secondary metabolites were also investigated using antiSMASH 7.0 [32]. A phylogenomic tree was generated on the type (Strain) genome server with the FastME 2.1.6.1 tool [33, 34].

### Morphology, Physiology, and Biochemical Analysis

The cell structure of the strain WR6^T^ was analyzed using transmission electron microscopy (Talos; FEI) after culturing on R2A agar at 25°C for three days, following previously described methods [35]. Gram-staining reactions were carried out with the Color Gram 2 kit (bioMérieux, France) following the manufacturer’s instructions. As described previously, catalase, oxidase, motility, and anaerobic growth ability were assessed [20]. The temperature, NaCl, and pH range for optimal growth were evaluated using previously illustrated methods [36]. Hydrolysis of DNA, CM-cellulose, casein, starch, and Tween 80 were analyzed following standard protocols [37]. Other enzymatic, biochemical, and carbon assimilation properties were determined using API ID 32 GN, API 20NE, and API ZYM kits (bioMérieux).

### Chemotaxonomic Characterization

Strain WR6^T^ and other closest taxa of the genus *Mesorhizobium* were cultured at 25°C on R2A agar for three days. Biomass was harvested and utilized for fatty acid extraction, analysis, and identification following the MIDI protocol [38]. Freeze-dried cells were prepared after growing strain WR6^T^ at 25°C on R2A agar for three days. Polar lipids and quinones were analyzed from freeze-dried cells [41, 42]. Polar lipid spots in the chromatograms were detected using various reagents [39].

## Results and Discussion

**Fig. 1:**
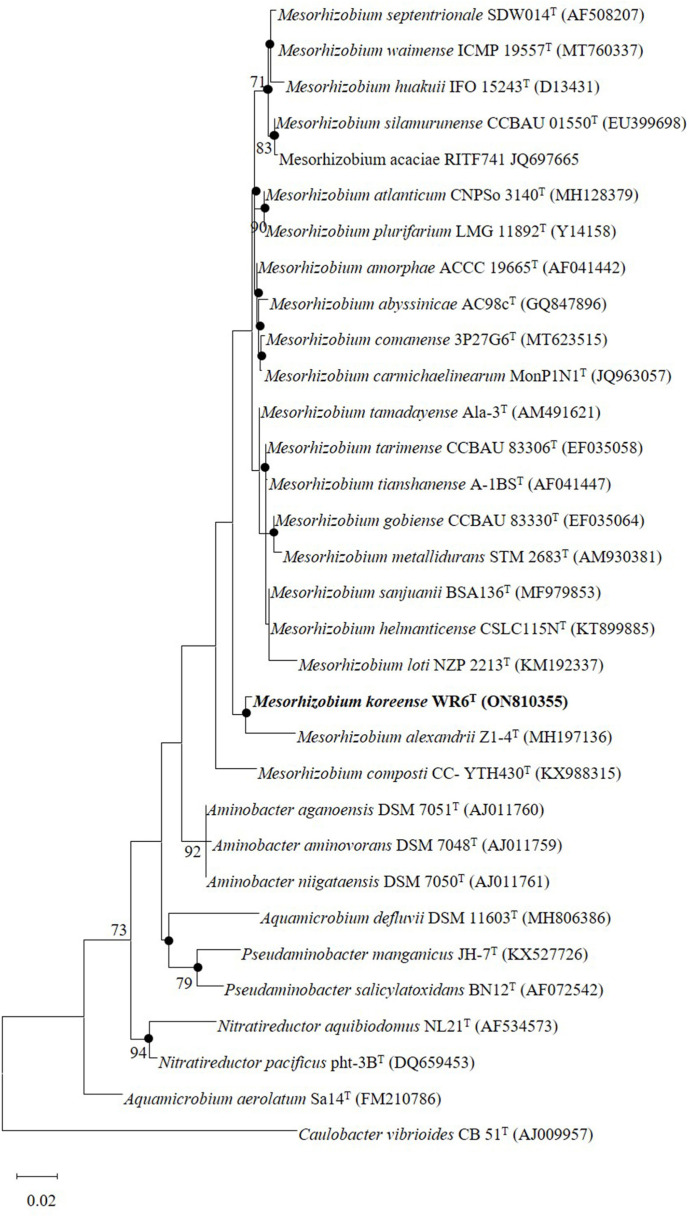
Maximum likelihood tree based on 16S rRNA gene sequences of strain WR6^T^ and closely affiliated reference taxa. Nodes recovered by maximum-likelihood and neighbor-joining trees are represented by filled circles. The numbers at branch nodes are percentage of 1,000 bootstrap replicates (values >70% are only illustrated). NCBI GenBank accession numbers for 16S rRNA gene sequences are provided in parentheses. *Caulobacter vibrioides* CB 51^T^ was used as an outgroup. The scale bar indicated 0.02 substitutions per nucleotide position.

The 16S rRNA gene nucleotide sequence of strain WR6^T^ was 1,416 bp. Analysis of the 16S rRNA gene nucleotide sequence revealed that strain WR6^T^ belonged to the genus *Mesorhizobium*, with the closest phylogenetic species being *Mesorhizobium waimense* ICMP 19557^T^ (98.5%). The 16S rRNA gene sequence similarities between strain WR6^T^ and all closest genetic neighbors were below the specified cut-off values of < 98.7%, as established for species demarcation [40, 41]. Both phylogenetic trees (ML and NJ) based on the 16S rRNA gene showed that strain WR6^T^ formed a clade with *Mesorhizobium alexandrii* Z1-4^T^ (Figs. 1 and S1), whereas phylogenetic trees (ML) based on the *gyrB* and *rpoB* genes generated a separate lineage for strain WR6^T^ within the members of the genus *Mesorhizobium* (Figs. S2 and S3).

The quality check confirmed the validity of the genome sequence of strain WR6^T^, displaying 92.0% completeness and 2.2% contamination. Strain WR6^T^ exhibited a genome size of 5,035,462 bp, with a DNA G+C content of 62.6%. The entire genome sequence of WR6^T^ was assembled into a single contig with a genome coverage of 124.0x (Table S1). RAST annotation revealed 338 subsystem features in strain WR6^T^ (Fig. S4). Analysis of biosynthetic gene clusters indicated the presence of genes encoding for ectoine, betalactone, and arylpolyene (Table S2). The dDDH and ANI values between strain WR6^T^ and its closest neighbors (*Mesorhizobium waimense* ICMP 19557^T^ and *Mesorhizobium amorphae* NBRC 102496^T^) ranged from 20.0% to 20.1% and 75.4% to 75.6%, respectively. These genomic relatedness values fell far below the established threshold values of 70.0% (for dDDH) [42] and 95.0% (for ANI) [42], indicating substantial genomic divergence of strain WR6^T^ from all other members of the genus *Mesorhizobium*. The phylogenomic tree illustrated that strain WR6^T^ formed distinct lineage within the genus *Mesorhizobium* (Fig. S5), consistent with the results of the phylogenetic tree analysis.

Cells of strain WR6^T^ were Gram-stain-negative, motile, rod-shaped, and flagellated (Fig. S6). Strain WR6^T^ exhibited growth at a temperature range of 10°C–30°C (optimum 25°C), a pH range of 5.0–8.5 (optimum 7.0), and a NaCl concentration of 3.0% (w/v) (optimum without NaCl). Catalase was positive, whereas oxidase was negative. Strain WR6^T^ was unable to hydrolyze esculin, starch, Tween 80, casein, DNA, urea, gelatin, and cellulose. Alkaline phosphatase, valine arylamidase, and α-glucosidase were negative, whereas trypsin, α-galactosidase, and β-galactosidase were positive for strain WR6^T^. The major distinguishing features of strain WR6^T^ are described in the species protologue and presented in Table 1 alongside the closest reference taxa. All data regarding enzyme activities and carbon assimilation properties resulting from API kits are displayed in Table S3.

The only ubiquinone detected in strain WR6^T^ was Q-8, similar to other species of the genus *Mesorhizobium* [12, 13]. Strain WR6^T^ contained diphosphatidylglycerol, phosphatidylglycerol, phosphatidylmethylethanolamine, phosphatidylcholine, and phosphatidylethanolamine. Additionally, unidentified aminolipids (AL1-AL2) were visualized (Fig. S7). The major polar lipids were similar to other species within the genus *Mesorhizobium* [10, 12]. The principal cellular fatty acids detected in strain WR6^T^ were summed feature 8 (C_18:1_*ω*7*c* and C_18:1_*ω*6*c*; 49.2%) and C_19:0_ cyclo *ω*8*c* (14.0%). The profile of principal cellular fatty acids resembles that of the closest species within the *Mesorhizobium* genus. However, the composition of minor fatty acids differed proportionally between strain WR6^T^ and the closest taxa (Table 2).

### Taxonomic Conclusion

In conclusion, the taxonomic data presented here confirm that the isolate WR6^T^ represents a novel species within the genus *Mesorhizobium* for which the name *Mesorhizobium koreense* sp. nov. is proposed.

### Description of *Mesorhizobium koreense* sp. nov.

*Mesorhizobium koreense* sp. nov. (ko.re.en’se. N.L. neut. adj. *koreense* pertaining to Korea).

Cells (4.2–4.3 × 2.4–2.5 μm) are aerobic, Gram-stain-negative, rod-shaped, flagellated, and motile. Colonies are white-coloured, circular, and convex with the diameter of 4.4–5.8 mm. Cells grow at temperature of 10–30°C (optimum 25°C), at pH of 5.0–8.5 (optimum 7.0), and at NaCl content of 0–3.0% (w/v) (optimum without NaCl). Cells are positive for catalase activity. Negative for oxidase, nitrate reduction, esculin, starch, Tween 80, casein, DNA, urea, gelatin, and cellulose hydrolysis. Positive for esterase (C4), esterase lipase (C8), leucine arylamidase, trypsin, acid phosphatase, naphtol-AS-BI-phosphohydrolase, α-galactosidase, and β-galactosidase. Assimilates L-arabinose, D-mannitol, *N*-acetyl-D-glucosamine, D-melibiose, D-sorbitol, propionate, 3-hydroxy-butyrate, L-proline, inositol, acetate, lactate, and glycogen. The only ubiquinone is Q-10; key cellular fatty acids are summed feature 8 (C_18:1_*ω*7*c* and/or C_18:1_*ω*6*c*) and C_19:0_ cyclo *ω*8*c*; and predominant polar lipids are diphosphatidylglycerol, phosphatidylglycerol, phosphatidylmethylethanolamine, phosphatidylcholine, and phosphatidylethanolamine. The DNA G+C content is 62.6%.

The type strain, WR6^T^ (=KCTC 92695^T^ =NBRC 116021^T^)., was isolated from soil samples collected from Republic of Korea (36°33'15.1"N 126°54'05.0"E).

The GenBank/EMBL/DDBJ accession numbers for the 16S rRNA nucleotide and genome sequences of strain WR6^T^ are ON810355 and CP134228, respectively.

## Supplemental Materials

Supplementary data for this paper are available on-line only at http://jmb.or.kr.



## Figures and Tables

**Table 1:** Differentiating properties of strain WR6^T^ and closely affiliated reference taxa.

Characteristic	1	2	3
Growth temperature (˚C)	10–30	15–30	10–35
Highest salt tolerance (%, w/v)	3.0	8.0	0.5
pH range	5.0–8.5	5.0–8.0	5.5–8.0
Catalase/oxidase	+/-	+/-	+/-
Esculin	-	-	-
Enzyme activity			
Alkaline phosphatase	-	+	+
Esterase Lipase (C8)	+	-	+
Valine arylamidase	-	-	-
Trypsin	+	-	-
*α*-Galactosidase	+	-	-
*β*-Galactosidase	+	-	-
*β*-Glucuronidase	-	+	-
*α*-Glucosidase	-	+	+
*β*-Glucosidase	-	-	-
Assimilation from			
(API 20NE and ID 32 GN test)			
D-Glucose	-	+	+
L-Arabinose	+	+	-
D-Mannose	-	+	+
D-Maltose	-	+	+
Malate	-	+	+
Citrate	-	+	-
Phenyl-acetate	-	+	-
D-Melibiose	+	+	-
L-Fucose	-	+	-
D-Sorbitol	+	+	-
Propionate	+	+	-
Valerate	-	-	-
3-Hydroxy-butyrate	+	-	-
L-Proline	+	-	-
L-Rhamnose	-	+	+
D-Ribose	-	-	-
Inositol	+	+	+
D-Sucrose	-	+	+
Acetate	+	-	-
Lactate	-	-	-
L-Alanine	-	-	-
Glycogen	+	-	-
L-Serine	-	-	-
DNA G + C content (%)	62.6	62.4	62.9

Strains: 1, WR6^T^; 2, *Mesorhizobium waimense* LMG 28228^T^; 3, *Mesorhizobium amorphae* NBRC 102496^T^. All data were obtained in this study. The data for G+C content was computed from genome sequences. +, positive; -, negative.

**Table 2:** Cellular fatty acid profiles of strain WR6^T^ and related reference members.

Fatty acid	1	2	3
Saturated			
C_10:0_	1.0	–	–
C_12:0_	1.0	–	tr
C_14:0_	2.2	–	tr
C_16:0_	8.3	14.0	12.4
C_17:0_	–	–	tr
C_18:0_	2.0	1.6	3.8
C_20:0_	–	–	tr
Unsaturated			
C_17:1_ *ω*6*c*	–	–	–
C_18:1_ *ω*9*c*	–	–	tr
C_20:1_ *ω*7*c*	–	–	tr
Branched saturated			
iso-C_10:0_	1.0	–	–
iso-C_13:0_	–	–	–
iso-C_13:0_ 3OH	–	–	tr
iso-C_15:0_	8.3	–	tr
iso-C_15:0_ 3OH	1.0	–	–
iso-C_17:0_	1.1	2.6	1.8
iso-C_18:0_	–	–	tr
anteiso-C_17:1_ *ω*9*c*	–	4.0	tr
C_10:0_ *ω*7*c* 11-methyl	–	3.2	5.6
Hydroxyl fatty acid	–	–	–
C_18:1_ 2OH	tr	–	–
Cyclo			
C_17:0_ cyclo	1.2	–	–
C_19:0_ cyclo *ω*8*c*	**14.0**	4.3	1.4
Summed features*			
3	9.2	2.3	tr
8	**49.2**	65.3	68.8

Strains: 1, WR6^T^; 2, *Mesorhizobium waimense* LMG 28228T; 3, *Mesorhizobium amorphae* NBRC 102496^T^. All data were obtained in this study. The data are presented in % of totals. TR, trace amount (<1.0%); –, not detected.

*Summed features represent groups of two or three fatty acids that could not be separated using the MIDI system. Summed feature 3 comprised C_16:1_*ω*7*c* and/or C_16:1_*ω*6*c* and Summed feature 8 comprised C_18:1_*ω*7*c* and/or C_18:1_*ω*6*c*. Unknown fatty acids are designated by their ECL, relative to the chain lengths of known straight-chain saturated fatty acids.
