# Supplementary material for: Mesorhizobium koreense sp. nov., Isolated from Soil
Source: J Microbiol Biotechnol. 2024 Jun 24;34(9):1819–25. doi: 10.4014/jmb.2404.04026 (PMC11473501; doi:10.4014/jmb.2404.04026)
Supplement: Supplementary file 1 [file jmb-34-9-1819-supple.pdf]

J. Microbiol. Biotechnol. 2024. 34(9): 1819-1825

https://doi.org/10.4014/jmb.2404.04026

Supplementary Tables and Figures

Mesorhizobium koreense sp. nov., Isolated from Soil

Hyosun Lee [1], Dhiraj Kumar Chaudhary [2], and Dong-Uk Kim [1]

[1] Department of Biological Science, College of Science and Engineering, Sangji University, Wonju, 26339, Republic of Korea

[2] Department of Microbiology, Pukyong National University, Busan, 48513, Republic of Korea

*Corresponding author: Dong-Uk Kim (dukim@sangji.ac.kr)

Table S1. The genome features of strain WR6^{T}.

**Genome features**	**WR6^{T}**
Genome size (bp)	5,035,462
G + C content (%)	62.6
No. of contigs	1
N50 (bp)	5,035,462
L50	1
No. of subsystem	338
No. of proteins	4,674
Total genes	4,787
CDSs (total)	4,733
Protein-coding genes	4,674
Genes (RNA)	47
rRNAs (5S, 16S, 23S)	1, 1, 1
Complete rRNAs (5S)	1, 1, 1
tRNAs	47
ncRNAs	4
Pseudo Genes (total)	59
Genome coverage	124.0x

Table S2. The distribution of biosynthetic gene clusters (BGCs) in the genome of the strain WR6^{T}.

WR6^{T}					
**Genomic regions**	**Type**	**From**	**To**	**Most similar known cluster**	**Similarity (%)**
Region 1	Ectoine	956,291	966,689	Ectoine	83
Region 2	Betalactone	2,271,276	2,303,060		
Region 3	Redox-cofactor	2,570,039	2,592,205		
Region 4	Terpene	3,545,387	3,566,226		
Region 5	Arylpolyene	3,741,468	3,782,646		

Table S3. The enzymatic and assimilation data obtained from API ZYM, API 20NE and API ID 32 GN tests of strain WR6^T and related reference members. Strains: 1, WR6^T; 2, *Mesorhizobium waimense* LMG 28228^T; 3, *Mesorhizobium amorphae* NBRC 102496^T. +, positive; -, negative.

**API ZYM test**	**1**	**2**	**3**
Alkaline phosphatase	-	+	+
Esterase (C4)	+	+	+
Esterase lipase (C8)	+	-	+
Lipase (C14)	-	-	-
Leucine arylamidase	+	+	+
Valine arylamidase	-	-	-
Cystine arylamidase	-	-	-
Trypsin	+	-	-
α-Chymotrypsin	-	-	-
Acid phosphatase	+	+	+
Naphtol-AS-BI-phosphohydrolase	+	+	+
α-Galactosidase	+	-	-
β-Galactosidase	+	-	-
β-Glucuronidase	-	+	-
α-Glucosidase	-	+	+
β-glucosidase	-	-	-
N–Acetyl -β-glucosaminidase	-	-	-
α-Mannosidase	-	-	-
α-Fucosidase	-	-	-
**API 20NE test**			
Reduction of nitrates (NO_3^-) to nitrites (NO_2^-)	-	-	-
Reduction of nitrates (NO_3^-) to nitrogen(N_2)	-	-	-
Indole production	-	-	-
Glucose Acidification	-	-	-
Arginine dihydrolase	-	-	-
Urease	-	-	-
β-Glucosidase (esculin hydrolysis)	-	-	-
Protease (gelatin hydrolysis)	-	-	-
β-Galactosidase (PNPG)	-	-	-
D-Glucose	-	+	+
L-Arabinose	+	+	-
D-Mannose	-	+	+
D-Mannitol	+	+	+
N-Acetyl-D-glucosamine	+	+	+
D-Maltose	-	+	+
Gluconate	-	-	-
Caprate	-	-	-
Adipate	-	-	-

Malate	-	+	+
Citrate	-	+	-
Phenyl-acetate	-	+	-
**API ID 32 GN test**			
D-Mannitol	+	+	+
D-Glucose	-	+	+
Salicin	-	-	-
D-Melibiose	+	+	-
L-Fucose	-	+	-
D-Sorbitol	+	+	-
L-Arabinose	+	+	+
Propionate	+	+	-
Caprate	-	-	-
Valerate	-	-	-
Citrate	-	+	-
L-Histidine	-	-	-
2-Ketogluconate	-	-	-
3-Hydroxy-butyrate	+	-	-
4-Hydroxy-benzoate	-	-	-
L-Proline	+	-	-
L-Rhamnose	-	+	+
N-Acetyl-D-glucosamine	+	+	+
D-Ribose	-	-	-
Inositol	+	+	+
D-Sucrose	-	+	+
D-Maltose	-	+	+
Itaconate	-	-	-
Suberate	-	-	-
Malonate	-	-	-
Acetate	+	-	-
Lactate	-	-	-
L-Alanine	-	-	-
5-Ketogluconate	-	-	-
Glycogen	+	-	-
3-Hydroxy-benzoate	-	-	-
L-Serine	-	-	-

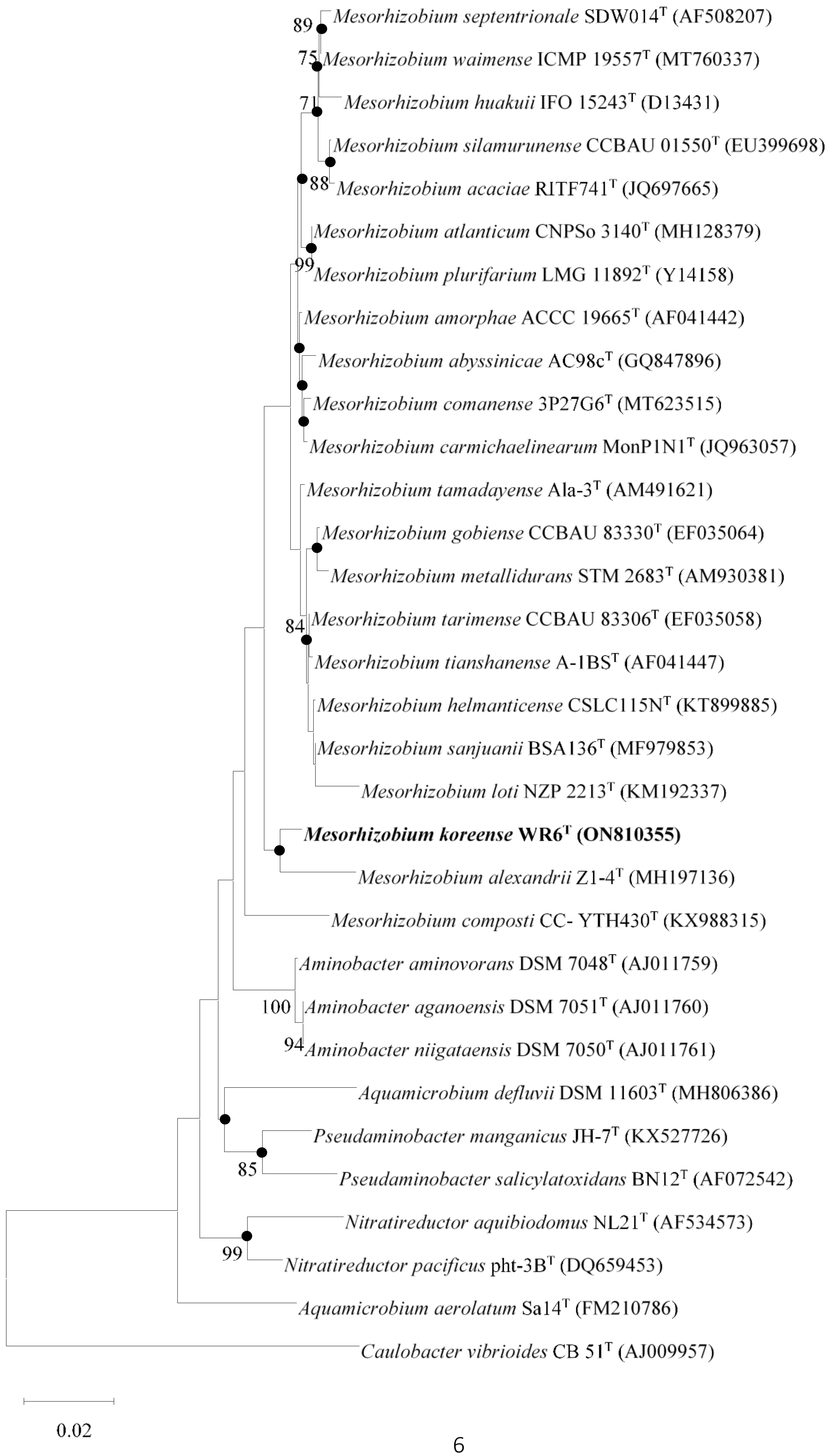
Mesorhizobium septentrionale SDW014T (AF508207)
Mesorhizobium waimense ICMP 19557T (MT760337)
Mesorhizobium huakuii IFO 15243T (D13431)
Mesorhizobium silamurunense CCBAU 01550T (EU399698)
Mesorhizobium acaciae RITF741T (JQ697665)
Mesorhizobium atlanticum CNPSo 3140T (MH128379)
Mesorhizobium plurifarium LMG 11892T (Y14158)
Mesorhizobium amorphae ACCC 19665T (AF041442)
Mesorhizobium abyssinicae AC98cT (GQ847896)
Mesorhizobium comanense 3P27G6T (MT623515)
Mesorhizobium carmichaelinearum MonP1N1T (JQ963057)
Mesorhizobium tamadayense Ala-3T (AM491621)
Mesorhizobium gobiense CCBAU 83330T (EF035064)
Mesorhizobium metallidurans STM 2683T (AM930381)
Mesorhizobium tarimense CCBAU 83306T (EF035058)
Mesorhizobium tianshanense A-1BST (AF041447)
Mesorhizobium helmanticense CSLC115NT (KT899885)
Mesorhizobium sanjuanii BSA136T (MF979853)
Mesorhizobium loti NZP 2213T (KM192337)
Mesorhizobium koreense WR6T (ON810355)
Mesorhizobium alexandrii Z1-4T (MH197136)
Mesorhizobium composti CC- YTH430T (KX988315)
Aminobacter aminovorans DSM 7048T (AJ011759)
Aminobacter aganoensis DSM 7051T (AJ011760)
Aminobacter niigataensis DSM 7050T (AJ011761)
Aquamicrobium defluvii DSM 11603T (MH806386)
Pseudaminobacter manganicus JH-7T (KX527726)
Pseudaminobacter salicylatoxidans BN12T (AF072542)
Nitratireductor aquibiodomus NL21T (AF534573)
Nitratireductor pacificus pht-3BT (DQ659453)
Aquamicrobium aerolatum Sa14T (FM210786)
Caulobacter vibrioides CB 51T (AJ009957)
89
75
71
88
99
84
100
94
85
99
0.02

36

Fig. S1. Neighbour-joining tree based on 16S rRNA gene sequences of strain WR6^T and closely affiliated reference taxa. Nodes recovered by maximum-likelihood and neighbor-joining trees are represented by filled circles. The numbers at branch nodes are percentage of 1,000 bootstrap replicates (values >70% are only illustrated). NCBI GenBank accession numbers for 16S rRNA gene sequences are provided in parentheses. *Caulobacter vibrioides* CB 51^T was used as an out-group. The scale bar indicated 0.02 substitutions per nucleotide position.

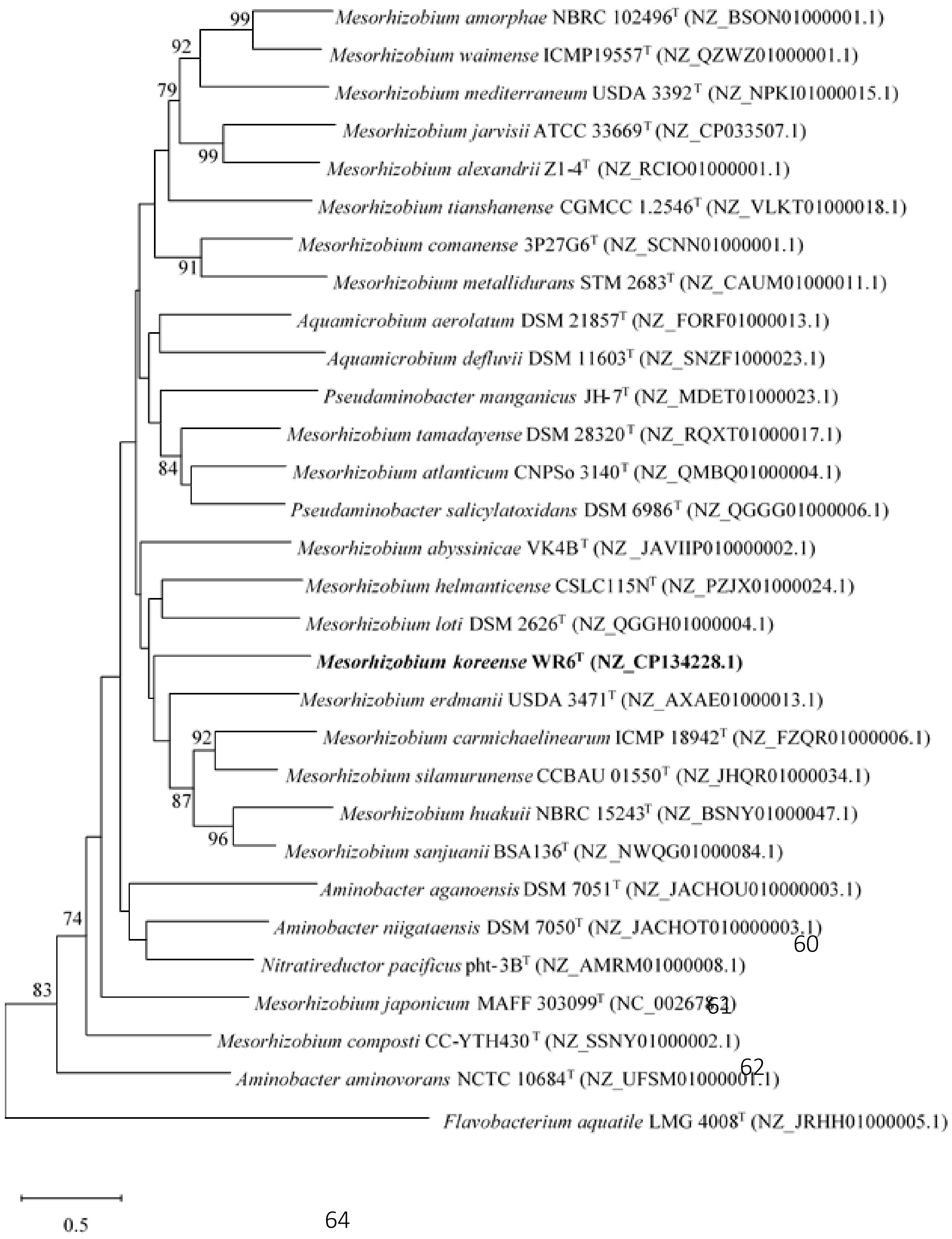

Fig. S2. Maximum likelihood tree based on *gyrB* gene sequences of strain WR6^T and closely affiliated reference taxa. The numbers at branch nodes are percentage of 1,000 bootstrap replicates (values >70% are only illustrated). NCBI GenBank accession numbers for *gyrB* gene sequences are provided in parentheses. *Flavobacterium aquatile* LMG 4008^T was used as an out-group. The scale bar indicated 0.5 substitutions per nucleotide position.

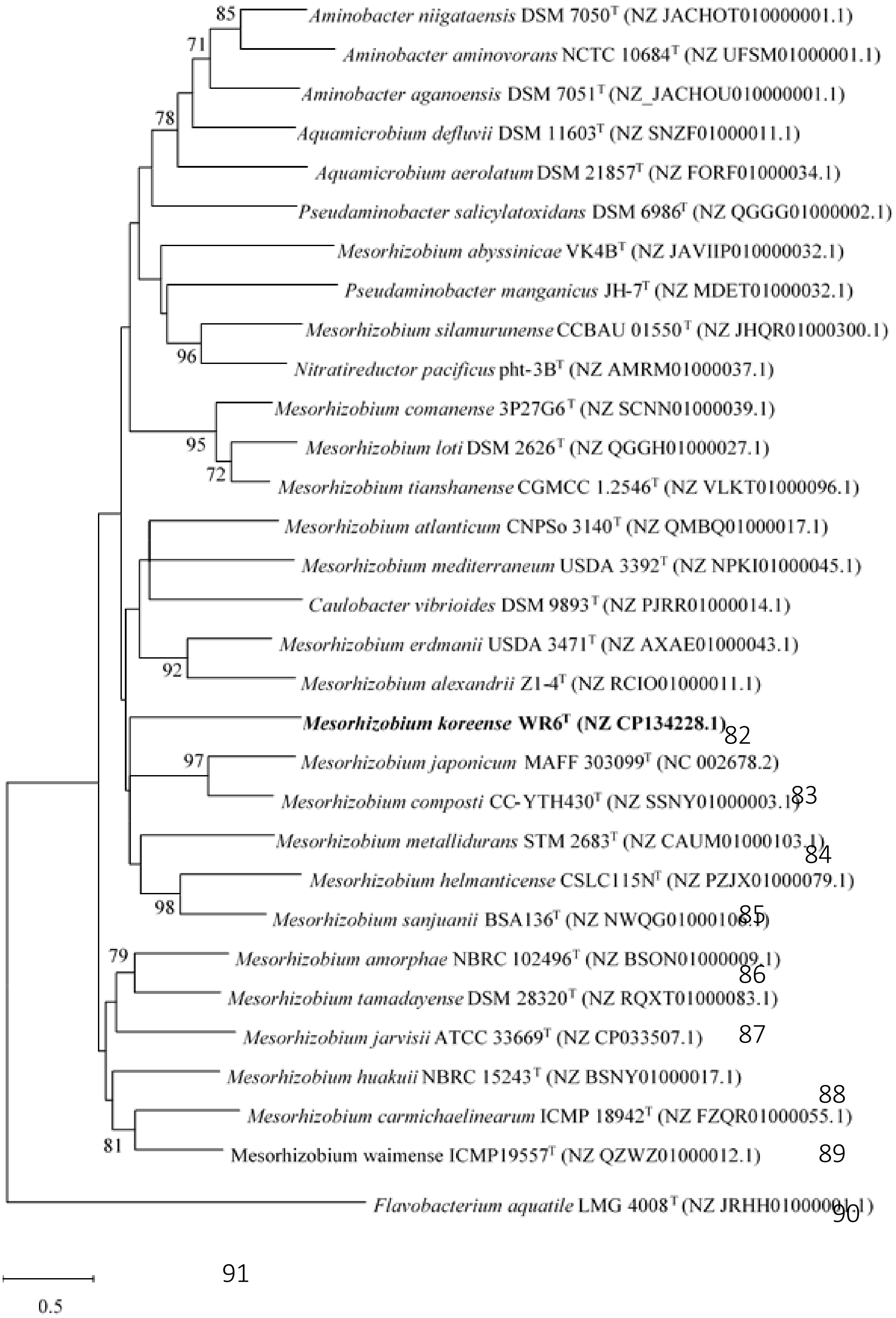

Fig. S3. Maximum likelihood tree based on *rpoB* gene sequences of strain WR6^{T} and closely affiliated reference taxa. The numbers at branch nodes are percentage of 1,000 bootstrap replicates (values >70% are only illustrated). NCBI GenBank accession numbers for *rpoB* gene sequences are provided in parentheses. *Flavobacterium aquatile* LMG 4008^{T} was used as an out-group. The scale bar indicated 0.5 substitutions per nucleotide position.

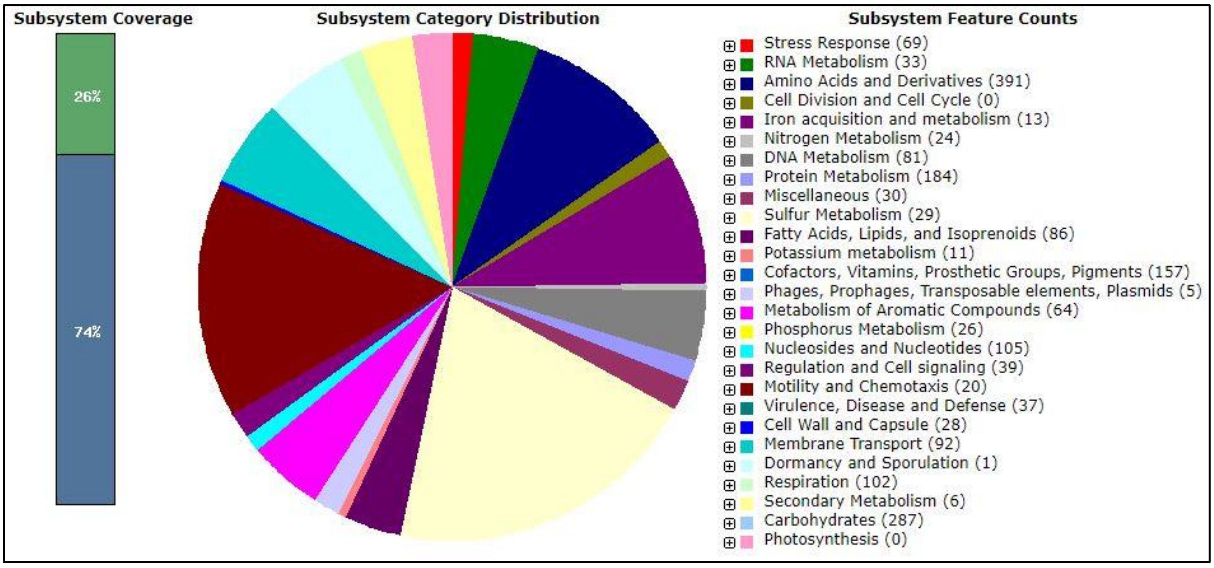

Fig. S4. Genome annotation of strain WR6^{T} performed by RAST (Rapid Annotation using Subsystem Technology) server.

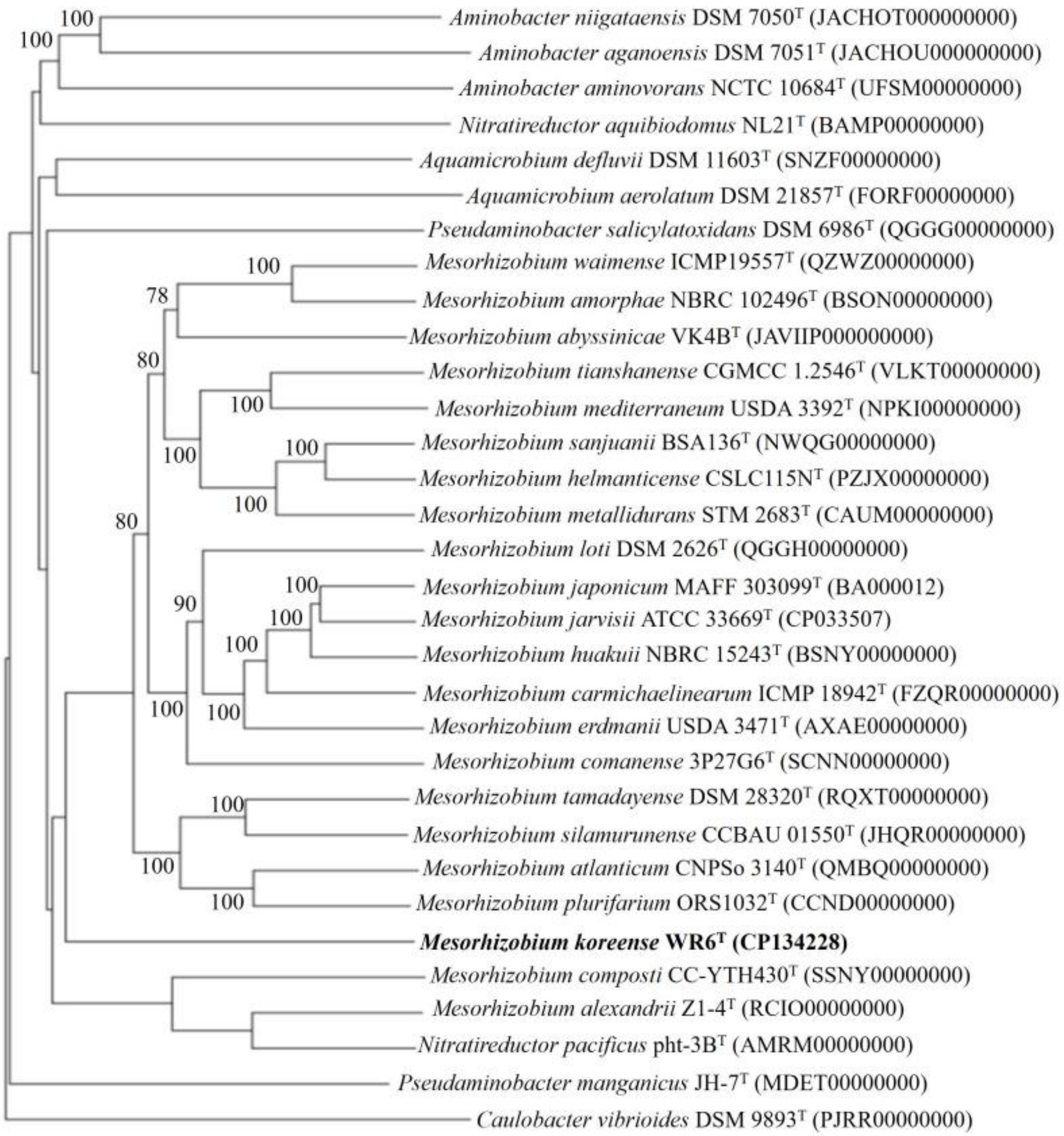

Fig. S5. Phylogenomic tree constructed with FastME 2.1.6.1 from GBDP distances calculated from genome sequence data. The numbers presented on the branches are GBDP pseudo-bootstrap support values from 100 replications.

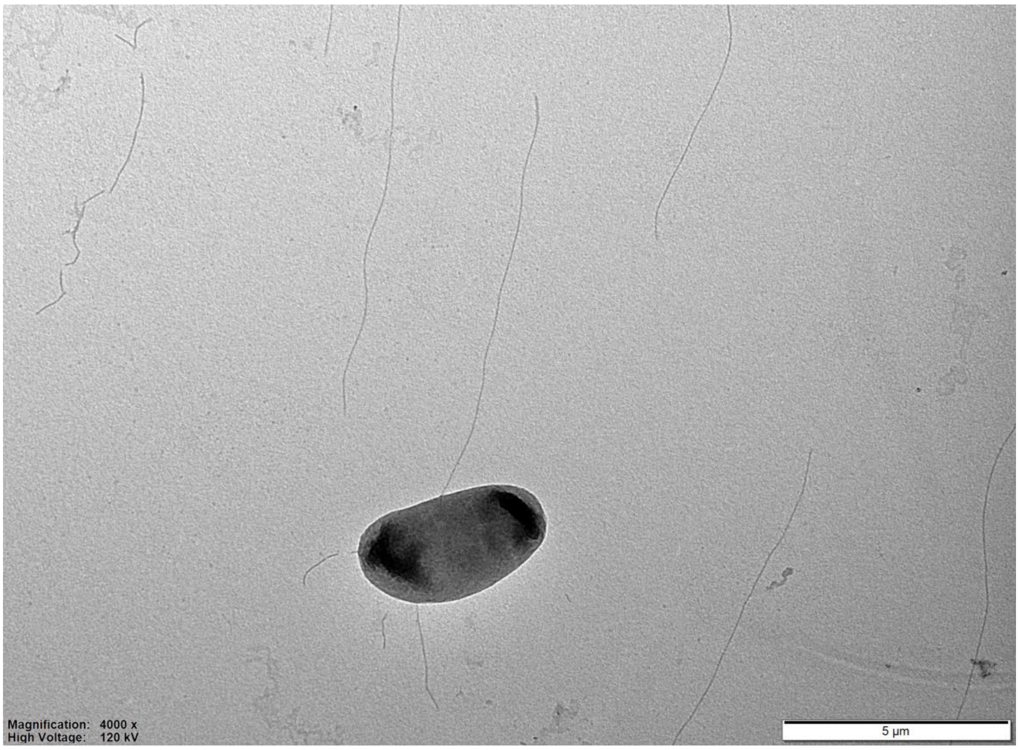

Fig. S6. Transmission electron photomicrograph of strain WR6^{T} grown on R2A agar for 3 days at 25°C. Bars, 5 µm.

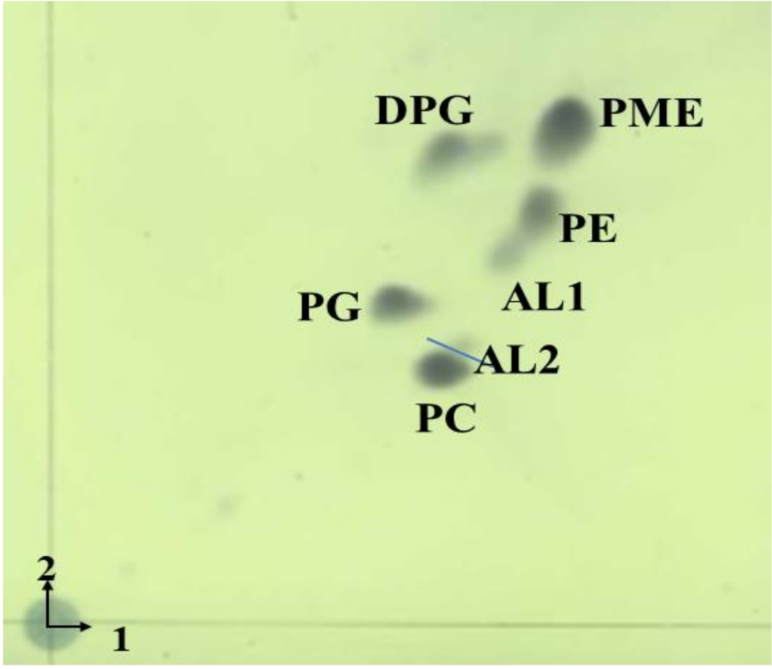

Fig. S7. Thin-layer chromatograms of the polar lipids from strain WR6^{T}. Abbreviations: DPG, Diphosphatidylglycerol; PG, Phosphatidylglycerol; PE, Phosphatidylethanolamine; PME, Phosphatidylmethylethanolamine; PC, Phosphatidylcholine; unidentified aminolipids (AL1-AL2).
